# Pyrolytic Conversion of Plastic Waste to Value-Added Products and Fuels: A Review

**DOI:** 10.3390/ma14102586

**Published:** 2021-05-16

**Authors:** Sadegh Papari, Hanieh Bamdad, Franco Berruti

**Affiliations:** Department of Chemical and Biochemical Engineering, Institute for Chemicals and Fuels from Alternative Resources (ICFAR), Western University, London, ON N6A 3K7, Canada; spapari@uwo.ca (S.P.); hbamdad@uwo.ca (H.B.)

**Keywords:** pyrolysis, plastic waste, carbon nanotubes, plastic oil, fuels, monomer recovery, olefins

## Abstract

Plastic production has been rapidly growing across the world and, at the end of their use, many of the plastic products become waste disposed of in landfills or dispersed, causing serious environmental and health issues. From a sustainability point of view, the conversion of plastic waste to fuels or, better yet, to individual monomers, leads to a much greener waste management compared to landfill disposal. In this paper, we systematically review the potential of pyrolysis as an effective thermochemical conversion method for the valorization of plastic waste. Different pyrolysis types, along with the influence of operating conditions, e.g., catalyst types, temperature, vapor residence time, and plastic waste types, on yields, quality, and applications of the cracking plastic products are discussed. The quality of pyrolysis plastic oil, before and after upgrading, is compared to conventional diesel fuel. Plastic oil yields as high as 95 wt.% can be achieved through slow pyrolysis. Plastic oil has a heating value approximately equivalent to that of diesel fuel, i.e., 45 MJ/kg, no sulfur, a very low water and ash content, and an almost neutral pH, making it a promising alternative to conventional petroleum-based fuels. This oil, as-is or after minor modifications, can be readily used in conventional diesel engines. Fast pyrolysis mainly produces wax rather than oil. However, in the presence of a suitable catalyst, waxy products further crack into oil. Wax is an intermediate feedstock and can be used in fluid catalytic cracking (FCC) units to produce fuel or other valuable petrochemical products. Flash pyrolysis of plastic waste, performed at high temperatures, i.e., near 1000 °C, and with very short vapor residence times, i.e., less than 250 ms, can recover up to 50 wt.% ethylene monomers from polyethylene waste. Alternatively, pyrolytic conversion of plastic waste to olefins can be performed in two stages, with the conversion of plastic waste to plastic oil, followed by thermal cracking of oil to monomers in a second stage. The conversion of plastic waste to carbon nanotubes, representing a higher-value product than fuel, is also discussed in detail. The results indicate that up to 25 wt.% of waste plastic can be converted into carbon nanotubes.

## 1. Introduction

Plastic products play a critical role in our lives and are being used in large quantities due to their durability, versatility, light weight, and low cost [1,2]. Plastic waste materials, generated in different sectors of the economy, such as agriculture, residential and commercial, automobiles, construction and demolition, packing materials, toys, and electrical equipment are growing rapidly and are either recycled, combusted (waste incineration), or disposed of [3]. Plastic waste consists mainly of low-density polyethylene (LDPE), high-density polyethylene (HDPE), polypropylene (PP), polyvinylchloride (PVC), polystyrene (PS), and polyethylene terephthalate (PET) [4]. Polyethylene and polypropylene constitute the greatest portion of plastic waste [4].

The increase in the world population and subsequent living standards have caused a rapid increase in municipal solid waste generation of to up to 1.3 billion tons per annum [3]. Reportedly, plastic waste is the third largest contributor of municipal solid waste [4]. The global production of plastic has increased, from 1.5 million tons in 1950 to approximately 359 million tons in 2018 and is attributed to a rapid rise in the packaging/wrapping sector [5]. Today, over 250 million tons per year are either landfilled or dispersed in the environment and an estimated 10 million tons per year end up in the oceans. Considering an increase of 9–13% of plastic waste per year [4], it is predicted that billions of tons of plastic could be produced by 2050, of which the greatest portion could go to landfills or be dispersed, both in the land environment and in the oceans.

An increase in daily demand of plastic materials, which are petroleum-based substances, can result in the depletion of non-renewable fossil resources. Approximately 4% of crude oil production is directly utilized in plastic production [6,7]. In addition to contributing to a global energy crisis, plastic waste can affect the environment and, therefore, either disposing or reusing/recycling is crucial. It is well known that plastics can persist in the environment for a prolonged period. The continuous disposal of plastic wastes is destructive to both terrestrial and marine ecosystem, as they are not readily biodegraded and can take several years to vanish [8]. Photo-degradation, auto-oxidation, thermo-oxidation, thermal degradation and biodegradation are plastic nature-based degradation mechanisms, however with a very slow rate [4]. On the other hand, the pollutants, such as toluenes, xylenes, benzenes, and phenols, released into the air, water, and soil as a result of plastic degradation cause undeniable issues, such as impacts on human and animal health and deterioration of soil fertility [4]. Photo-degradation converts plastic waste into fine pieces (micro-plastics), which float on the surfaces of rivers, ponds, lakes, end up in seas and oceans and can penetrate into the food chain and subsequently pass to humans [9].

In addition to landfilling, there are four distinctive approaches for plastic waste management. Primary mechanical recycling is a technique in which single-type uncontaminated and clean plastic wastes are reprocessed, resulting in a product without changing the basic structure and equivalent quality. Although this method is cost-effective, washing the waste materials generates a new waste stream and, more importantly, plastic wastes usually consist of mixtures of different plastics, often arranged as composites with other materials and are either difficult or impossible to recycle. Secondary recycling is another mechanical recycling approach that follows a decontamination process, remelting, remolding and re-extruding. Size reduction, contaminants removal, separation from other waste materials, make this approach less favorable in terms of operating cost. Tertiary recycling is a chemical or/and thermochemical recycling, which includes chemolysis/solvolysis (i.e., glycolysis, hydrolysis, methanolysis, and alcoholysis), gasification, partial oxidation, and pyrolysis. In this approach, large polymer molecules of plastic wastes are converted into shorter molecules through the use of heat and/or chemical reactions. This technique produces fuels or value-added chemicals that are useful for the synthesis of new plastics and other products. Quaternary recycling is waste combustion (incineration) of the waste material for energy recovery. It seems to be the simplest method; however, it generates pollution and may not meet the circular economy milestones [10,11]. 

According to the waste management hierarchy (from the most to the least preferred), we identify prevention, minimization, reuse, recycle, energy recovery, and disposal. Energy recovery through incineration is in a lower rank compared to reuse and recycling [12]. As mentioned, the primary and secondary recycling methods suffer some drawbacks, such as labor-intensive operation for the separation process prior to recycling, a high material loss, possible water contamination, and, overall, a high cost. More importantly, the recycled products are often more expensive than the virgin plastics and may not maintain the original properties. As such, the thermochemical conversion (i.e., tertiary recycling) can be an economically and environmentally friendly solution, leading to a high value fuel/chemical production from plastic waste.

The interest in thermochemical conversion of plastic waste, particularly pyrolysis, has increased considerably over the last few years, primarily since China stopped accepting post-consumer plastic waste in 2018, after having taken up to 45% of the world’s plastic waste for recycling, landfilling and incineration [13,14,15,16]. Zhang et al. [17] conducted a comprehensive review on various advanced non-biodegradable plastic waste treatment technologies. They concluded that physical recycling methods are the most sustainable technologies with consideration of a decrease in the performance of plastic after several recycling cycles. It has been stated that pyrolysis is the most widely used thermal remediation, and gasoline/diesel yield is an indicator parameter which can reflect the actual valuable yield of the process and its industrial applicability. Degradation is another promising technology, but most studies focus on using selected kinds of microorganism to degrade specific polymers. As such, research on microorganisms able to degrade mixtures of various plastics is needed. Fojt et al. [18] critically reviewed the overlooked challenges associated with the accumulation of micro-plastics in the soil. Products made from biodegradable plastics are beginning to replace conventional plastics. Composting is highly suggested for bioplastic disposal; however, the compost formed could contain micro-bioplastic particles resulting from incomplete biodegradation, causing soil contamination. These authors addressed this problem by summarizing sample pre-treatments and analytical techniques. The analytical techniques include both thermo-analytical (i.e., pyrolysis) and non-thermo-analytical (i.e. pre-sorting and respective detection limits) approaches. They concluded that, due to the poor knowledge of the production rate of micro-plastics, fate, sorption properties and toxicity, a rapid and suitable approach is required for their determination. Yet, thermo-analytical approach is the most promising strategy. Murthy et al. [19] carried out an in-depth review study of the plastic pyrolysis process and discussed the influence of various operating parameters as well as the characterization of the liquid oil obtained from the process. The results revealed that most of the plastics produce oil with reasonable calorific values (i.e., approximately similar to conventional fuels). The plastic pyrolysis product distribution depends on the type of reactor used. Significant studies have been conducted on batch-style reactors due to the easy design, fabrication, operation, and control. On the other hand, continuous fluidized bed reactors can provide a uniform mixing of feedstock and heat carriers or catalysts during operation and, therefore, generate more stable products. Nanda and Berruti [20] systematically reviewed solid waste technologies, such as pyrolysis, liquefaction and gasification for converting waste plastic into fuels/chemicals. They stated that pyrolysis and hydrothermal liquefaction technologies are able to reduce the volume of plastics to landfills/oceans, reduce the overall carbon footprints, and, more importantly, have high conversion efficiencies and relatively lower costs when compared to higher temperature processes, such as gasification. Selectively, plastics can be converted either to bio-oil, bio-crude oil, synthesis gas, hydrogen and aromatic char. As such, the influence of process parameters, such as temperature, heating rate, feedstock concentration, reaction time, reactor type, and catalysts, have been discussed thoroughly. Damodharan et al. [21] conducted a review on the utilization of waste plastic oil in diesel engines. They used waste plastic oil obtained from the pyrolysis of mixed waste plastics in the presence of catalyst (e.g. silica, alumina, ZSM-5 and kaolin), with up to 80 wt.% yield. The pyrolysis oil had a lower cetane number than fossil diesel and, therefore, longer ignition delays and higher heat releases. NOx emissions are higher with plastic pyrolysis oil. Smoke emissions were chiefly low with plastic oil and could be further decreased to Euro levels by the use of oxygenated additives. They finally concluded that, plastic pyrolysis oil is a good candidate for fossil diesel replacement and found it to run smoothly in diesel engines. Williams [22] carried out a review on converting waste plastic to hydrogen and carbon nanotubes via pyrolysis coupled with catalytic steam reforming. This author investigated the influence of reactor designs, catalyst type, and operating conditions on the yield and quality of the carbon nanotubes. He concluded that the process temperature along with the type of catalyst are the prominent factors in plastic to hydrogen and carbon nanotubes pyrolysis. There is a balance between introduction of steam, which enhances hydrogen yield, and carbon nanotubes quality, since higher steam flowrates tend to oxidize the carbon nanotubes.

This review aims to thoroughly discuss the different types of plastic waste pyrolysis processes (i.e., slow, fast, and flash) with respect to the quality and quantity of the products. Furthermore, the application of pyrolysis plastic oil, as a fuel and/or material, is reviewed. Upgrading plastic oil through different methods (e.g., thermal cracking for monomer recovery, hydrogenation, and blending) along with the conversion of plastic waste to carbon nanotubes is reviewed and discussed in detail. This paper contributes to the science of waste management and waste valorization, providing the most updated information and insight through a comprehensive study of the most advanced literature on the pyrolytic conversion of waste plastics.

## 2. Plastic Waste Properties

To achieve a very good heat/mass transfer during the pyrolysis process, plastic wastes are typically crushed, shredded and sieved to obtain small size flakes, i.e., less than 2 mm. Proximate and ultimate analysis of different plastic wastes are presented in Table 1 and Table 2. A high volatile matter content (above 90 wt.%) along with a high carbon and hydrogen content make plastic waste an excellent candidate for the pyrolysis process, leading to a high conversion to the liquid/gas products.

## 3. Pyrolysis Process

Pyrolysis is a versatile thermal cracking process that occurs in the absence of oxygen at temperatures above 400 °C. Typically, pyrolysis processes are classified as slow, fast, and flash [24,25]. This thermochemical process breaks down the long chain polymer molecules into smaller and less complex molecules through heat and chemical reactions. Slow pyrolysis is typically performed at temperatures between 350 and 550 °C, with 1 to 10 °C/min heating rates, and a prolonged vapor residence time. The major product of slow pyrolysis is a solid residue, called char, as a slow heating rate favors solid formation among various parallel-competitive reactions [25]. Fast pyrolysis often takes place at temperatures between 500 and 700 °C. The heating rate experienced by the feedstock is above 1000 °C/min, and vapor residence times are normally in the range of a few seconds [26]. Fast pyrolysis favors liquid production and, depending on the feedstock type, the liquid yield can surprisingly reach up to 90 wt.% for the pyrolysis of polyolefin materials [27]. In flash pyrolysis, the temperature is usually above 700 °C, the heating rate experienced by the feed is extremely fast, and vapor residence times are in the range of milliseconds [25]. Flash pyrolysis can produce higher yields of oil than fast pyrolysis for biomass feedstocks, while it differs for plastic waste, as the latter produces more gas compared to other products [28]. The products obtained from the pyrolysis of plastic wastes (all types, alone, or as mixtures) are categorized into liquid/wax, solid residues, and gas [29].

Pyrolysis is a robust technique and can be used for either fuel or monomer recovery, particularly while addressing plastic waste management challenges. For example, liquid pyrolysis oil obtained from fast pyrolysis is an excellent source of gasoline and diesel [29]. Unlike water-rich pyrolysis bio-based oils derived from biomass feedstocks, the plastic oils have a high heating value, almost three times more than bio-oils, and similar to diesel fuels, due to absence of highly oxygenated compounds and water [30]. The acid content of plastic oils also is dramatically lower than that of bio-oils and, therefore, no further upgrading may be required for fuel applications. The pyrolysis coproducts include solid (char), which can be used as an adsorbent [31,32,33], and gas, a valuable resource that can be used as an energy supplier for the pyrolysis process.

The produced plastic oil can be a liquid or a wax. The wax is yellowish with a high viscosity at room temperature and is predominantly composed of alkanes and alkenes hydrocarbons with a high boiling point (C20+) [34]. Wax is typically an intermediate product, and a further process, such as fluid catalytic cracking (FCC), is required to convert it into liquid fuels. Liquid plastic oil is comprised of mainly aliphatic compounds as well as mono- and polyaromatics [29]. In addition to being a promising precursor for fuel applications, the plastic oil can be used as an intermediate and converted into ethylene and propylene through further cracking at higher temperatures and extremely low contact times. The major gaseous species forming the “gas product” are methane, ethylene, ethane, propylene, butadiene, and butane [35]. The gas product can be used as an energy source to provide the required pyrolysis energy, making the process self-sustaining and independent from external energy sources. In addition, the valuable olefin components present in the gas stream can be separated and recovered for chemical recycling. The solid residue is the remaining pyrolysis product, mostly made of coke and ash [36].

### 3.1. Slow Pyrolysis

Table 3 summarizes slow pyrolysis trials conducted for the conversion of plastic wastes with/without catalyst utilization using various reactors and under a wide range of operating conditions. The results reveal that the liquid produced during slow pyrolysis is typically oily rather than waxy. The oil yield can reach up to 93 wt.% when LDPE is pyrolyzed at 550 °C, which implies a remarkable yield with a broad range of applications [21]. The plastic oil is versatile and can be used either directly in steam boilers for electricity generation or as a platform chemical for other applications, such as transportation fuels, monomer recovery, and carbon nanotubes (CNTS) production. The solid residue yield is significantly less than bio-based char, as a consequence of the lower fixed carbon and a higher volatile matter associated with plastic wastes compared to biomass (Table 1). The gasoline fraction, C6–C12, can constitute up to 90 wt.% of the liquid product, making it valuable for conventional gasoline replacement.

#### 3.1.1. Influence of Plastic Types

Table 3 indicates that the pyrolysis of polyolefins, including LDPE, HDPE, and PP, typically produces a liquid oil with a significant fraction of aliphatic (alkanes and alkenes), specifically in the absence of catalyst. The impact of catalyst on the composition of plastic oil is discussed later, in Section 3.1.2. The desired pyrolysis temperature to achieve a high conversion of polyolefins is above 450 °C, since, below this temperature, the solid residue drastically increases. Polystyrene (PS), which is composed of styrene monomers, can generate a liquid with a remarkable amount of aromatic compounds, such as benzene, toluene, and ethyl benzene [2]. Although the pyrolysis of polyolefins and polystyrene leads to the formation of a liquid oil which can be an excellent precursor for fuels/chemicals, the pyrolysis of PET and PVC generates a significant amount of benzoic acid and hydrochloric acid, respectively, which are toxic and corrosive to the reactors [2,37]. As such, these two polymers are typically excluded from pyrolysis.

#### 3.1.2. Influence of Catalyst

Among the typical plastic pyrolysis process catalysts (e.g., FCC, HZSM-5, MCM-41, HY, Hβ, HUSY, mordenite and amorphous silica-alumina), acidic zeolites have been widely investigated [38,39,40,41,42,43]. Zeolite catalysts have shown an excellent catalytic efficiency on cracking, isomerization and oligomerization/aromatization, attributed to their specific physicochemical properties, including a strong acidity and a micropore crystalline structure [44]. As illustrated in Table 3, the plastic pyrolysis in the presence of catalysts, particularly HZSM5, tends to produce remarkably more aromatic and polycyclic aromatic hydrocarbons, compared to the uncatalyzed pyrolysis process, therefore contributing to the gasoline fraction. Further, a significantly higher production of gases is typically observed in the presence of zeolite catalysts, due to the enhanced cracking reactions [45]. Amorphous silica-alumina catalysts significantly contribute to the production of light olefins, with no noticeable changes in the aromatics formation [46]. ZSM-5 and zeolite-Y promote the formation of both aromatics and branched hydrocarbons, along with a significant increase in the proportion of gaseous hydrocarbons. These results are consistent with other published reports in the literature [47,48]. Catalytic reforming over Al-MCM-41 proactively contributes to the gasoline production with a lower impact on gas generation, likely due to the weaker acid properties and larger pore dimensions of the catalyst [1]. In the presence of both Y-zeolite and ZSM-5 catalysts, the oil yield dramatically decreases in favor of gas production [49]. The superiority of Y-zeolite compared to the ZSM-5, in terms of aromatic compounds production, is rationalized by the differences in physical and chemical catalyst properties, i.e., pore size, surface area, and surface acidity [50].

### 3.2. Fast Pyrolysis

Unlike slow pyrolysis, which is typically performed in the batch reactors, fast pyrolysis takes place in continuous systems. The faster char removal from the reactor space associated with continuous processes prevents undesirable catalytic effects leading to the excessive cracking of vapors, which, coupled with short vapor residence times, minimizing secondary cracking reactions, result in a higher liquid production. Table 4 indicates that fast pyrolysis can convert up to 95 wt.% of plastic waste into the liquid/wax product (e.g., pyrolysis of HDPE at 600 °C). In addition, PE yields a higher liquid/wax product compared to the PP. The waxy fraction (C20+) of fast pyrolysis is greater than that of slow pyrolysis, attributed to a shorter vapor residence time and, consequently, reduced cracking reactions. As previously mentioned, the waxy product, which is an excellent source of paraffins and olefins, can be used as a feedstock in FCC units for the production of transportation fuels and other valuable petrochemical compounds.

#### Influence of Temperature

Temperature plays a key role in all pyrolysis processes, regardless of the feedstock type. In the pyrolysis of plastic wastes, as in any other pyrolysis process, the increase in temperature results in a rapid increase in gas yields from the enhanced cracking reactions and, correspondingly, in a decrease of the oil/wax yield (Table 4). In addition to the alteration of yields, temperature expectedly affects the products quality, due to its impacts on the pyrolysis kinetic mechanisms. Generally speaking, high temperature favors the production of less waxy and more oily compounds production, attributed to the conversion of long-chain paraffins/olefins to shorter molecules. Conversely, the solid residue yield decreases at elevated temperatures. A qualitative assessment of plastic oil shows high temperature favors an increase in gasoline production corresponding to a higher concentration of aromatics [52]. The yield of ethylene and propylene are found increase as the temperature rises.

### 3.3. Flash Pyrolysis

In order to avoid over-cracking reactions during pyrolysis, especially at high temperatures (above 700 °C), which converts a significant amount of liquid to gaseous products, flash pyrolysis taking place within milliseconds is a suitable option. Unlike the fast pyrolysis of biomass, which generates the highest yields of bio-oil, flash pyrolysis of plastic waste produces more gas rather than liquids (Table 5). As illustrated in Table 5, up to 75 wt.% of monomers, i.e., ethylene and propylene, can be recovered through flash pyrolysis. The by-product, which is oil, can be used to provide the required energy for the process. Kannan et al. [56] performed a flash pyrolysis of LDPE in a microreactor with a minimal heat/mass transfer resistance at temperatures of 600–1000 °C, and vapor residence time of 250 ms to investigate the effect of temperature on monomer recovery (yield of olefins). They found that the 950–1000 °C temperature range is optimal to recover up to 68 wt.% of monomers.

#### Influence of Vapor Residence Time

**Figure 1:**
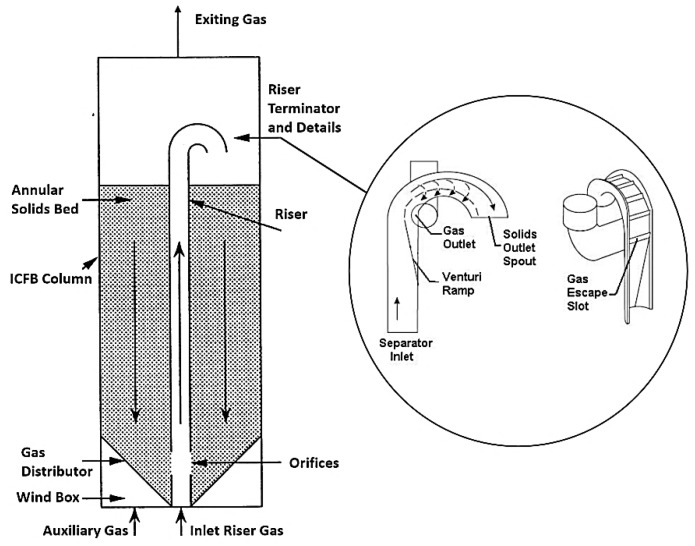
Internally circulating fluidized bed (ICFB) and riser terminator (adapted from [58,60]).

Together with temperature, the vapor residence time in the hot zone (i.e. reactor) is the key parameter that significantly affects the pyrolysis products yields and compositions. Previous work [28,57,58] on the flash pyrolysis of different waste plastics in micro and large scale reactors indicated that short vapor residence time resulted in a high olefin content gas. Although the Internally Circulating Fluidized Bed (ICFB) reactor illustrated in Figure 1 has been shown to be suitable for thermochemical processes requiring short residence times, this capability is not achieved without cost [58]. The control of the residence time, particularly for large scale reactors, presents a significant challenge to the reactor designer. Nielsen et al. [60] invented a fenestrated centrifugal riser terminator for use in an ICFB and/or in conventional fluid bed reactor risers. This terminator can separate solids from gas in less than 20 ms with 99.5% separation efficiency. Such a fast separation is critically important when the objective is to minimize the vapor residence time at high temperature and rapidly quench the reaction (Table 5).

## 4. Plastic Oil Cracking

**Figure 2:**
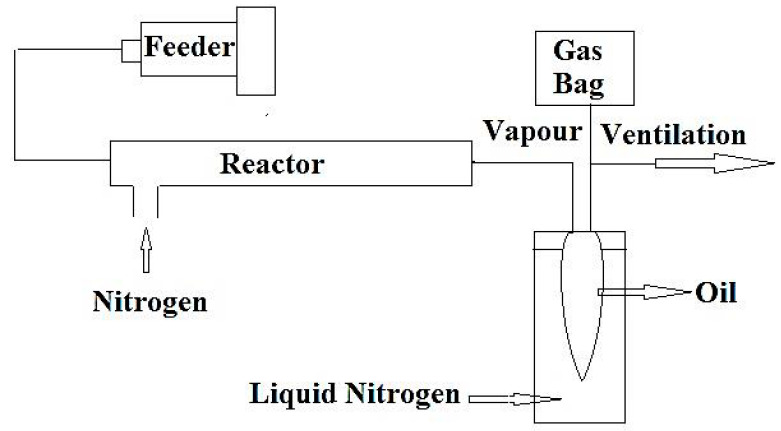
Schematic diagram of the experimental apparatus for cracking of raw plastic pyrolysis oil and extracted oil after separation of aromatics (adapted from Reference [35]).

Tsuji et al. [35] utilized a two-stage unit, including a liquid–liquid extraction followed by a pyrolysis reactor, to investigate the thermal cracking of pyrolysis plastic oils containing considerable amounts of aromatic compounds, such as styrene. In the first stage, sulfolane solvent was used to remove the aromatic compounds prior to the pyrolytic cracking, in order to mitigate the coking effects, since stable aromatics (e.g., styrene) tend to be coked rather than cracked during pyrolysis. The results were promising, and the gas yield reached 85 wt.% at 750 °C, corresponding to a 20 wt.% increase compared to non-extracted oil. A schematic diagram of the pyrolysis oil cracking set-up is shown in Figure 2. 

The results of cracking of different plastic oils are summarized in Table 6. SL and SM are the light and medium fractions of pyrolysis plastic oil obtained from Sapporo Plastic Recycle Co. DH and DL stand for heavy and light fractions of plastic oil obtained from Dohoh Recycle Center Co. in Japan. Model plastics waste oil was obtained in the lab [35] from the pyrolysis of mixed plastics at 450 °C. The analysis of data presented in Table 6 suggests that a higher cracking temperature (above 850 °C) and a lower vapor residence time (less than 730 ms) are required to potentially achieve the best monomer recoveries.

## 5. Upgrading of Pyrolysis Plastic Oils

The comparison between diesel fuel and a sample plastic oil obtained from the pyrolysis of mixed plastics, including 58 wt.% of HDPE and LDPE, 27 wt.% of PP, 9 wt.% of PS, 5 wt.% of PET [61] is shown in Table 7. The GC-MS results reveal that the carbon number distributions of the produced plastic oil is as follows: 35.41 wt.% of C6–C9, 48.40 wt.% of C10–C14, 13.21 wt.% of C15–C20, and 1.83 wt.% of C20+ [61]. The comparison between fuel properties of pyrolysis plastic oil and diesel indicates similar heating values and kinematic viscosity, while the plastic oil has a higher ash content and a lower cetane number compared to diesel fuel.

### 5.1. Blending

As discussed earlier, raw pyrolysis plastic oil has a high heating value (40–55 MJ/kg), a low water content (<1 wt.%), and approximately neutral pH. Therefore, boilers can readily burn it as-is for the electricity generation. Damodharan et al. [21] stated that diesel engines can smoothly run plastic oil and no modification is required to the existing engine infrastructure. In contrast, there are several researchers [8,62,63,64] who believe improvements in plastic oil quality are needed to meet EN590 standards. In terms of drawbacks associated with the utilization of pyrolysis plastic oil in internal combustion engines, a high heat release and delayed ignition have been reported [61]. As such, a blend of conventional fuel and plastic oil can be a potential solution. The fuel trials using blends in conventional engines reveal a stable performance with less emission of NOx and SOx compared to diesel and gasoline fuels [65]. A reduced specific fuel consumption has also been reported [61]. Awasthi and Gaikwad [66] stated that the overall performance of the blend of diesel and plastic oil in a single cylinder four stroke VCR diesel engine was very satisfactory, particularly with 20 wt.% of pyrolysis plastic oil. Singh et al. [61] experimentally showed that the blend of plastic oil with diesel up to 50% can be easily utilized in conventional diesel engines.

### 5.2. Hydrogenation

Hydrogenation process takes place in the presence of three components: hydrogen, a catalyst, and an unsaturated compound. The transfer of hydrogen pairs to the unsaturated compound is facilitated via a heterogeneous catalyst which enables the reaction to occur at a lower temperature and pressure. For instance, hydrogenation converts alkenes to alkanes in plastic oil [67]. Due to the significant presence of unsaturated compounds in the plastic oils, some storage instability challenges may be experienced over time. Hydrogenation of pyrolysis oil occurring at temperatures above 700 °C, pressures around 70 bar, and in the presence of catalyst (such as ZSM-5) can alter unsaturated compounds into saturated and makes the oil more stable. A combination of hydrogenating and blending have been suggested to upgrade the plastic oil quality in order to meet the EN590 standard [67]. The fuel properties of plastic oil, diesel, and hydrogenated plastic oil along with the EN590 standard are compared in Table 8.

### 5.3. Liquid-Liquid Extraction

The high aromatic content of plastic oils, particularly those obtained from pyrolysis of mixed plastics containing polystyrene, leads to a decrease in the engine performance and an increase in emissions due to a long ignition delay [68]. Generally, the low cetane number fuels, caused by high aromatic content, are not suitable for conventional diesel engines as they can cause unstable combustion. As such, the aromatic compounds can be separated and removed via solvent extraction prior to the utilization of plastic oil in diesel engines. Sulfolane as solvent was proven effective by Tsuji et al. [35] for the separation of aromatic compounds.

## 6. Carbon Nanotubes

**Figure 3:**
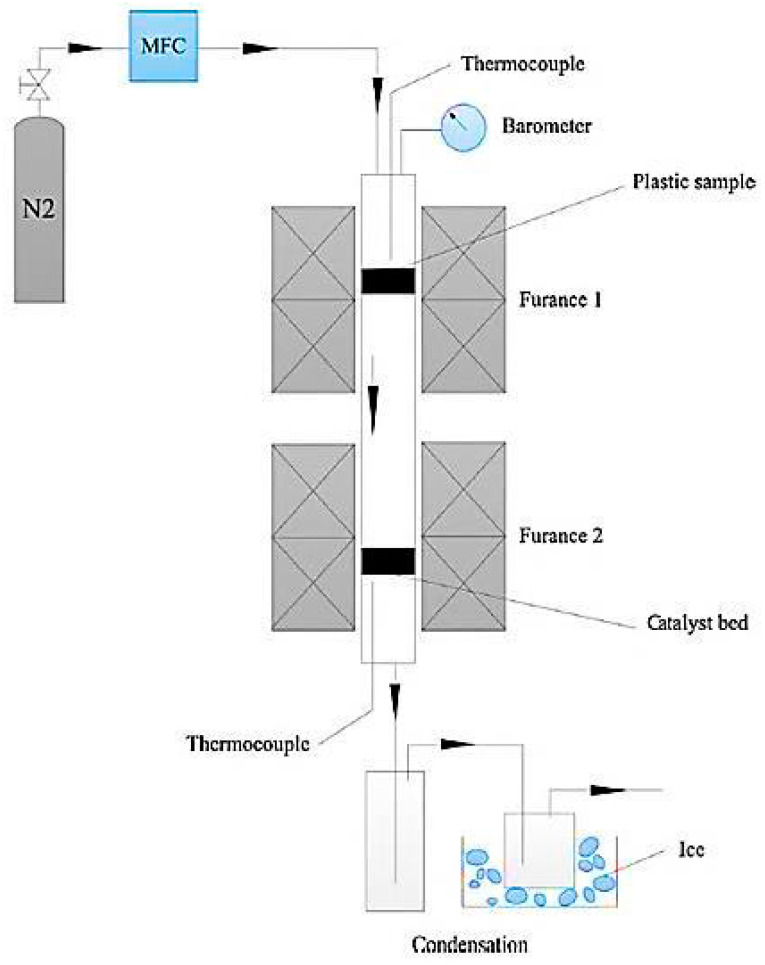
Schematic diagram of two-stage pyrolysis reactor system (adapted from [73]).

**Figure 4:**
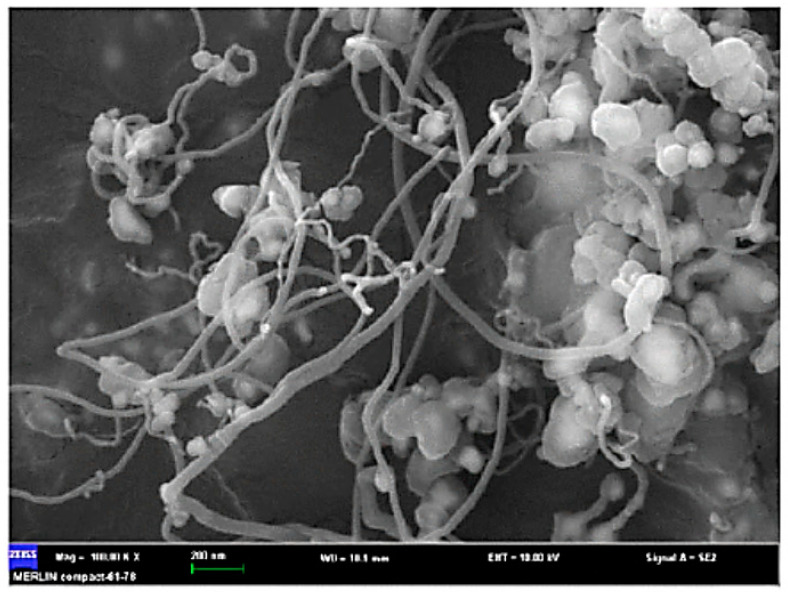
CNT growth on Ni-based catalyst (adapted from [73]).

Carbon nanotubes (CNTs) have gained recognition as very attractive materials due to their unique properties, including great electrical conductivity (100 times greater than copper), excellent mechanical strength (100 times greater than steel), high thermal conductivity, stable chemical properties, extremely high thermal stability, and an ideal one-dimensional (1D) structure with anisotropy [69,70,71,72]. Conventionally, methane, natural gas, acetylene, and benzene from nonrenewable resources have been utilized as a feedstock for CNTs production. Recently, the potential fabrication of CNTs from the pyrolysis of plastic waste has drawn researchers’ attentions, adding a significant value to the plastic wastes. The process of converting plastic waste into CNTs is composed of two successive stages (Figure 3). In the first stage, the plastic waste is converted to the volatile vapor in the absence of oxygen and at a moderate temperature (approximately 550 °C). Then, the produced vapor is introduced into the second stage at a high pressure of 1 MPa and a temperature of 750 °C in the presence of Ni-based catalyst where it is converted into CNTs on the surface of the catalyst through the chemical vapor deposition mechanism. In this advanced process, CNT yields can reach up to 25 wt.% [73]. In the second stage, during pyrolysis at 750 °C, plastic waste vapors further decompose to the mixtures of their monomers (e.g., ethylene, propylene, and styrene). These light gases serve as carbon donors for CNTs formation. Moreover, the produced vapors from the first stage contain a significant amount of hydrogen, which plays an undeniable role in the formation of CNTs. Hydrogen moderates the rate of carbon deposition and prevents catalyst deactivation and poisoning by continuous surface cleansing of the catalyst surfaces [69,70,71,72]. A SEM image of CNT growth on Ni-based catalyst is shown in Figure 4.

## 7. Conclusions

The pyrolytic conversion of plastic waste into value added products and/or fuels is extensively reviewed in this paper. Plastic waste, which can be a source of detrimental problems to terrestrial and marine ecosystems, can be thermochemically converted into valuable products, such as gasoline, diesel, and wax. Fast pyrolysis leads to the production of waxy hydrocarbon mixtures, whereas slow pyrolysis typically produces more oil than wax. This is attributed to a difference in the vapor residence times, since longer residence times in slow pyrolysis allow for more cracking reactions breaking down the larger molecules into smaller and lighter fragments. The utilization of catalyst in plastic pyrolysis favors aromatic compounds in the liquid phase and gas production. Higher pyrolysis temperatures result in enhanced secondary cracking reactions, and, therefore, in a greater conversion of waxy compounds to oily and gaseous products. PE and PP produce pyrolysis oils with more aliphatic compounds, while PS generates higher aromatic hydrocarbons. In flash pyrolysis, conducted at 1000 °C and 250 ms of vapor residence time, up to approximately 70 wt.% of olefin monomers including 50 wt.% of ethylene can be recovered, making it a promising process for monomer recovery. The plastic oil can be blended with diesel and utilized as a fuel in conventional diesel engines. A two-stage process, including a pyrolysis unit followed by a fixed bed reactor with a nickel-based catalyst can be utilized to convert up to 25 wt.% of plastic waste into very valuable carbon nanotubes.

## Figures and Tables

**Table 1:** Ultimate analysis of different plastic wastes [23].

Plastic Types	Carbon	Hydrogen	Oxygen	Nitrogen	Sulfur
HDPE	78	13	4	0.06	0.08
PP	84	14	1	0.02	0.08
PS	90	9	1	0.07	0.08
PET	77	13	5	0.20	NA

**Table 2:** Proximate analysis of various plastic types [2].

Plastic Types	Moisture Content	Fixed Carbon	Volatile Matters	Ash Content	HHV (MJ/Kg)
HDPE	0.0	0.3	99.8	1.4	49.4
LDPE	0.3	0.0	99.7	0.4	46.4
PP	0.2	1.2	97.8	1.9	46.4
PS	0.3	0.2	99.6	0.0	41.9
PET	0.5	7.8	91.8	0.1	30.2

**Table 3:** Slow pyrolysis of different plastic wastes.

Plastic Types, Temp., Cat., Ref.	Feed: Catalyst Ratio	Liquid/Wax Yield (wt.%)	Solid Residue Yield (wt.%)	Gas Yield (wt.%)	Gasoline (C6–C12) (wt.%)	Diesel (C13–C20) (wt.%)	Wax (C20+) (wt.%)	Monomer Recovery (wt.%)
HDPE-450 °C-None-[46]	-	84	3	13	47	32	5	-
HDPE-450 °C-ZSM-5-[46]	20	35	2	63	35	0	0	-
HDPE-450 °C-Silica-alumina-[46]	20	78	1	21	71	7	0	-
LDPE-425 °C-None-[1]	10	44	45	11	20	24	<1	3
LDPE-450 °C-None-[1]	10	74	10	16	34	39	1	7
LDPE-475 °C-None-[1]	10	69	4	27	28	36	<1	16
LDPE-425 °C-HZSM-5-[1]	10	7	48	45	7	<1	<1	26
LDPE-450 °C-HZSM-5-[1]	10	16	11	73	15	0	1	47
LDPE-475 °C-HZSM-5-[1]	10	22	4	74	21	<1	<1	53
LDPE-475 °C-Al-MCM-41-[1]	10	40	50	10	30	7	1	7
LDPE-475 °C-Al-MCM-41-[1]	10	34	18	58	31	3	2	31
LDPE-475 °C-Al-MCM-41-[1]	10	42	4	54	38	5	1.5	37
PE-500 °C-Y-zeolite-[49]	NR	80	10	10	NR	NR	NR	4
PE-500 °C-ZSM-5-[49]	NR	70	10	20	NR	NR	NR	5
LDPE-550 °C-None-[21]	10	93	-	14	NR	NR	NR	9
HDPE-550 °C-None-[45]	10	84	-	16	NR	NR	NR	11
LDPE-550 °C-LDPE-HZSM5-[45]	10	18	1	71	NR	NR	NR	59
HDPE-550 °C-LDPE-HZSM5-[45]	10	17	1	72	NR	NR	NR	53
LDPE-550 °C-HUSY-[45]	10	62	2	34	NR	NR	NR	22
LDPE-550 °C-HUSY-[45]	10	41	2	39	NR	NR	NR	31
HDPE-450 °C-None-[50]	34	74	19	6	15	60	25	21
HDPE-450 °C-MCM-[50]	34	78	15	6	15	60	25	22
HDPE-450 °C-FCC-[50]	34	82	11	6	25	65	10	21
HDPE-450 °C-HZSM-5-[50]	34	81	4	15	25	62	23	21
PS-550 °C-[51]	-	90	2	9	42	37	11	3
PET-550 °C-None-[51]	-	84	4	12	-	-	-	-
Mixed-550 °C-None-[51]	-	83	6	11	56	20	6	4

**Table 4:** Fast pyrolysis of different plastic wastes.

Plastic Type, Temp., Ref.	Liquid/Wax Yield (wt.%)	Solid Residues Yield (wt.%)	Gas Yield (wt.%)	Gasoline (C6–C12) (wt.%)	Diesel (C13–C20) (wt.%)	Wax (C20+) (wt.%)	Monomer Recovery (wt.%)
PP-668 °C-[52]	43	2	54	40	-	-	26
PP-703 °C-[52]	35	6	57	34	-	-	27
PP-746 °C-[52]	29	4	65	29	-	-	17
PE-728 °C-[52]	38	2	59	36	-	-	34
HDPE-600 °C-[53]	95	-	5	18	25	53	4
HDPE-650 °C-[53]	85	-	15	27	21	37	12
HDPE-700 °C-[53]	60	-	40	32	17	11	37
HDPE-428 °C-[53]	93	-	7	52	33	17	-
PP-409 °C-[53]	96	-	4	70	21	9	-
HDPE-650 °C-[54]	80	-	20	10	18	52	-
PVC-740 °C-[55]	28	49	15	-	-	-	-

**Table 5:** Flash pyrolysis of LDPE with different experimental parameters.

Plastic Type, Temp., Ref.	Vapor Residence Time (s)	Liquid/Wax Yield (wt.%)	Solid Residues Yield (wt.%)	Gas Yield (wt.%)	Monomer Recovery Yield (wt.%)
LDPE-900 °C-[57]	0.75	-	-	95.0	50
LDPE-850 °C-[54]	0.6	11.4	-	88.6	-
LDPE-825 °C-[58]	0.4	5	2	93	75
LDPE-790 °C-[59]	0.5	32.1	0.2	62.2	51.6
LDPE-1000 °C-[56]	0.25	-	-	99	68

**Table 6:** Pyrolysis of different plastic wastes [35].

Plastic Type and Temp.	Vapor Residence Time (s)	Liquid/Wax Yield (wt.%)	Solid Residues Yield (wt.%)	Gas Yield (wt.%)	Ethylene Yield (wt.%)	Propylene Yield (wt.%)	Total Olefin Yield (wt.%)
SL-700 °C	0.96	43.1	0.1	28.4	7	7	16
SL-850 °C	0.81	34.6	3.9	31.1	15	18	20
SM-700 °C	0.91	30.1	2.1	49.4	15	25	30
SM-850 °C	1.06	26.2	4	-	25	30	35
DL-700 °C	0.95	31.4	0.2	46.2	10	20	25
DL-850 °C	0.77	28.6	2.2	41.8	20	25	30
DH-700 °C	0.95	32.1	2	54.3	20	15	40
DH-850 °C	0.75	18.6	2.3	65	40	5	50
MO-700 °C	0.92	26.7	0.8	45	15	10	32
MO-850 °C	0.73	28.8	2.7	55.3	30	5	38

**Table 7:** A comparison between plastic oil and diesel physicochemical properties [61].

Properties	Plastic Oil *	Diesel
Density (kg/m^3^)	734	820–850
Ash content (wt.%)	1	0.04
Calorific value (MJ/kg)	41	42
Kinematic viscosity (cSt)	2.9	3.05
Cetane number	49	55
Flash point (°C)	46	50
Fire point (°C)	51	56
Carbon residue (wt.%)	0.01	0.002
Sulphur content (wt.%)	<0.001	<0.035
Pour point (°C)	−3	−15
Cloud point (°C)	−27	-
Aromatic content (wt.%)	32	11–15

* Composition: 35.41 wt.% of C6-C9, 48.40 wt.% of C10-C14, 13.21 wt.% of C15-C20, and 1.83 wt.% of C20+.

**Table 8:** Physicochemical properties of diesel, plastic oil and hydrogenated plastic oil [67].

Properties	Lower Limit Standards EN590	Upper Limit Standards EN590	Diesel	Plastic Pyrolysis Oil	Hydrogenated Plastic Pyrolysis Oil
Density (kg/m^3^)	820	840	837	771	851
Pour Point (°C)	-	-	−15	−30	−20
Flash Point (°C)	55	-	72	20	65
Fire Point (°C)	-	-	82	30	72
Calculated Cetane Index	46	-	52	60	62
Kinematic Viscosity (mm^2^/s)	2	4.5	2.31	1.78	3.5
Gross Calorific Value (MJ/kg)	-	-	46	45	45
Ash Content (wt.%)	-	0.1	0.01	0.01	0.01
Conradson Carbon Residue (wt.%)	-	-	0.18	0.1	0.1

## Data Availability

Data sharing not applicable.
